# An Adaptive Method for Gait Event Detection of Gait Rehabilitation Robots

**DOI:** 10.3389/fnbot.2020.00038

**Published:** 2020-07-17

**Authors:** Jing Ye, Hongde Wu, Lishan Wu, Jianjun Long, Yuling Zhang, Gong Chen, Chunbao Wang, Xun Luo, Qinghua Hou, Yi Xu

**Affiliations:** ^1^Shenzhen MileBot Robotics Co., Ltd., Shenzhen, China; ^2^Shenzhen Institute of Geriatrics, Shenzhen, China; ^3^Department of Rehabilitation Medicine, The Seventh Affiliated Hospital, Sun Yat-sen University, Shenzhen, China; ^4^Rehabilitation Center, The First Affiliated Hospital of Shenzhen University, Shenzhen, China; ^5^Stroke Biological Recovery Laboratory, Spaulding Rehabilitation Hospital, Harvard Medical School, Boston, MA, United States; ^6^School of Medical Instrument and Food Engineering, University of Shanghai for Science and Technology, Shanghai, China; ^7^Kerry Rehabilitation Medicine Research Institute, Shenzhen, China; ^8^Shenzhen Sanming Project Group, Spaulding Rehabilitation Hospital, Harvard Medical School, Boston, MA, United States; ^9^Shenzhen Dapeng New District Nan'ao People's Hospital, Shenzhen, China; ^10^Clinical Neuroscience Center, The Seventh Affiliated Hospital, Sun Yat-sen University, Shenzhen, China

**Keywords:** gait event detection, inertial sensor, adaptive threshold, gait rehabilitation robot, adaptive method

## Abstract

Accurate gait event detection is necessary for control strategies of gait rehabilitation robots. However, due to personal diversity between individuals, it is a challenge for robots to detect a gait event at various stride frequencies. This paper proposes a novel method for gait event detection of a gait rehabilitation robot using a single inertial sensor mounted on the thigh. A self-adaptive threshold for detecting heel strike is obtained in real time via a linear regression model. Observable thresholds for toe off detection are constant at various stride frequencies. Experiments are conducted based on 20 healthy subjects and six hemiplegic patients wearing a gait rehabilitation robot and walking at various kinds of stride frequencies. The experimental results show that the proposed method can detect heel strike and toe off gait events within an average 2% gait cycle temporal errors and never miss two-gait event detection. Compared to the conventional thresholding method, this work presents a simple and robust application for gait event detection in healthy and hemiplegic subjects by one inertial sensor. The linear regression model can be applicable to different subjects walking at various stride frequencies.

## Introduction

In the field of gait rehabilitation robots, synchronization between motion of robots and actual human gait is very important. This requires that the robot can accurately identify the current gait event, and then it automatically adjusts its gait phase to achieve human–robot synchronization. Besides, a gait cycle of human walking consists of a stance phase and a swing phase (Perry, 2010). There are different control strategies for the two phases of gait rehabilitation robots. For example, during a stance phase, stability and high damping are requisite, and for a swing phase, high velocity and low damping are necessary (Ledoux, 2018). The stance and swing phases are separated by heel strike (HS) and toe off (TO) gait events. Accurate HS/TO gait event detection decides which control strategy to use for gait rehabilitation robots; otherwise, it is likely to cause harm to patients.

More recently, many algorithms based on the inertial measurement unit (IMU) have been proposed to identify gait events. Thresholding is the most simple and practical method, and various algorithms for gait event detection have been developed (Mansfield and Lyons, 2003; Sabatini et al., 2005; Jasiewicz et al., 2006; Lau and Tong, 2008; Lau et al., 2008; Hanlon and Anderson, 2009; Anna and Wickström, 2010; Catalfamo et al., 2010; Kotiadis et al., 2010; Rueterbories et al., 2010; Varol et al., 2010; Tao et al., 2012; Goršič et al., 2014; Ledoux, 2018). Ledoux developed a thresholding method for gait event detection of human walking using a single IMU mounted on the shank (Ledoux, 2018). This method detected gait events using a set of fixed observable thresholds of shank angle, shank angular velocity, and axial acceleration. It can detect 100% of HS/TO gait events within an average of 2% gait cycle error for both healthy subjects and amputees. Catalfamo et al. proposed a fixed thresholding method for ambulatory gait analysis (Catalfamo et al., 2010). In this method, mean difference between the proposed method and the reference was <25 ms for HS and <75 ms for TO. Detection success was over 98%. However, the fixed thresholds are not adaptive regulation.

In addition, with the improvement of computational efficiency, machine learning methods have been used to detect gait events. Some groups have implemented gait event detection via hidden Markov models (HMM) or Gaussian mixture models (Mannini and Sabatini, 2010, 2012, 2013; Wang et al., 2010; Bae and Tomizuka, 2011). For instance, Mannini and Sabatini. presented a classifier based on an HMM model. The model was applied to identify four adjacent gait events by using a uniaxial gyroscope that measured foot instep angular velocity in the sagittal plane (Mannini and Sabatini, 2012). Bae and Tomizuka. described an HMM model for six gait phases detection (Bae and Tomizuka, 2011). Smart shoes embedded four air bladder–type force sensors that were utilized to obtain the ground reaction forces as observed data in the HMM. However, these methods based on an HMM model are used to detect adjacent gait events, and they do not identify non-adjacent gait events (such as HS and TO) in time sequence.

The above researches are most focused on information analysis from sensors mounted on the shank or foot. The reason is that foot signals can directly reflect gait event information, and shank signals are less variable between subjects (Wu, 1995; Tong et al., 1998; Catalfamo et al., 2010). However, we find that, compared to the thigh, the motion of the shank and foot have higher speed and variable acceleration while walking, which may cause higher accumulated error when calculating the shank or foot angle based on angular velocity detected by IMU. For humans, the thigh segment plays an important role for transmitting force to walk a step, and an IMU sensor mounted on the thigh is always required to detect various human motion intentions (Hornero et al., 2013; Lewis and Sahrmann, 2015; Borghetti et al., 2017). For patients with hemiplegia, monitoring the thigh angle obtained from an IMU sensor can detect abnormal postures and quantify patients' inabilities. If HS and TO gait events are also detected by an IMU sensor fixed on the thigh, it will make the sensor system simple and inexpensive. Thus, it is necessary to propose a method for gait event detection via one IMU sensor mounted on the thigh. Besides, Borghetti et al. shows that ranges of thigh angle have significant differences between different subjects (Borghetti et al., 2017). The fixed thresholds for gait event detection cannot meet the requirements of high accuracy. They need to be adjusted adaptively in different subjects and various stride frequencies. If the adaptive thresholds can achieve adequate real-time accuracy for walking, the method for gait event detection will become more convenient and robust in the practical application for gait rehabilitation robots. In this paper, the gait rehabilitation robot that refers to BEAR-H1 is self-developed in order to help patients with hemiplegia conduct rehabilitation training.

The focus of this paper is to build a linear regression model, which can detect an HS gait event for different subjects walking at various stride frequencies. Besides, it obtains a set of observable fixed thresholds for TO gait event detection. The proposed method is based on only one three-axis IMU mounted on the thigh of BEAR-H1. The IMU provides three signals: thigh angle, thigh angular velocity, and forward axial acceleration. The linear regression (LR) model and the set of observable fixed thresholds are achieved by the data set from 20 healthy subjects and six patients with hemiplegia. The data set shows that the HS gait event happens after the thigh angle peak. HS is detected when the current thigh angle is below the threshold obtained via the LR model, whose inputs are the three signal values at the thigh angle peak. TO is detected when the current three signal values are within the set of fixed thresholds. Accuracies of the proposed method are validated against two pressure sensors fixed in insoles.

The rest of this paper is organized as follows: Section Method for Gait Event Detection briefly introduces the data acquisition process and describes the proposed method for HS and TO detection based on the adaptive thresholding method and observable thresholds. Section Experiments presents experiments in hemiplegic subjects and results from the proposed method and the conventional thresholding method in healthy and hemiplegic subjects. Section IV is the discussion, and this paper ends with a conclusion in Section Conclusion.

## Method for Gait Event Detection

**Figure 1:**
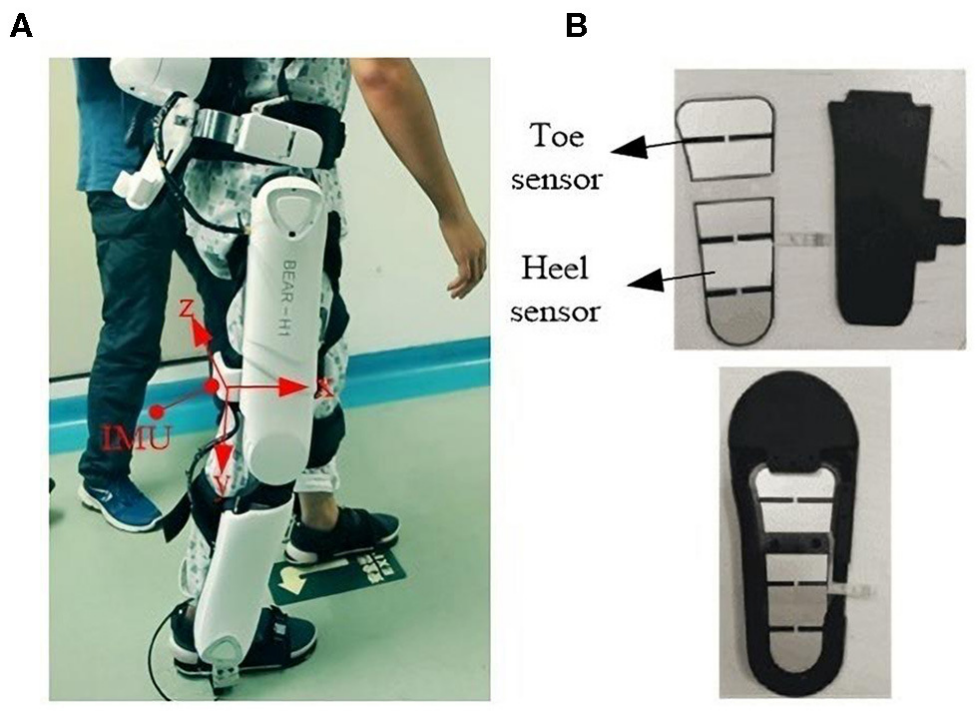
Data acquisition system. **(A)** A subject wearing the BEAR-H1 robot; **(B)** two pressure sensors.

The conventional thresholding method for gait event detection is based on fixed thresholds (Catalfamo et al., 2010; Ledoux, 2018). The proposed method adopts adaptive thresholds for HS detections and observable thresholds for TO detections. In this paper, three signals are used for the gait event analysis: thigh angle θz, thigh angular velocity ζz, and forward axial acceleration ax. The coordinate system is presented in Figure 1A. In this section, first, the data acquisition system and data acquisition process for 20 healthy subjects are described. Second, based on these data, the sharing characteristics for HS and TO gait events between different subjects are summed up, and then, according to these characteristics, the adaptive thresholding method for HS gait event detection and an observable thresholding method for TO gait event detection are proposed. Finally, model training and parameter selection are discussed in detail.

### Data Acquisition System

A gait rehabilitation robot is selected to acquire the data for gait event detection. The robot named BEAR-H1 is self-developed, which is used to help patients with hemiplegia do rehabilitation training. It has three training modes, including training mode, weight loss mode, and intelligent mode. The training mode is a passive mode, and the others are active modes. For training mode, stride frequency can be changed within 3% of the set gait cycle frequency. For intelligent mode, stride frequency can be adjusted in real time to achieve synchronization of human–robot interaction.

The robot has three active degrees of freedoms (DOFs) and a passive DOF in each leg. The three DOFs are rotations along the hip joint, knee joint, and ankle joint in the sagittal plane, separately. They are actuated by motors. The adduction and abduction of the hip joint is a passive DOF. Each thigh of the robot has an IMU (YIS100-V), shown in Figure 1A. Two pressure sensors are fixed in insoles of the robot at the toe and the heel, shown in Figure 1B. The sensors are used to validate the accuracy of the proposed method.

Specifically, the angle in the sagittal plane between thigh segment and vertical direction is denoted as the hip joint angle. There is a vertical standing calibration for the lower extremity exoskeleton robot before using. During the calibration process, the robot is hanging on a holder, and two thigh segments are parallel with the vertical direction, and that is the standing state (as the neutral position). In this state, hip joint angle is recorded as 0°. The walking process is begun at the standing state, and real-time hip joint angles are calculated by accumulating the product of angular velocity and Δt, where angular velocity is given by gyroscopes in real time and Δt is sampling interval.

### Data Acquisition and Protocol

In order to obtain the sharing characteristics for HS and TO gait events between different subjects, 20 healthy subjects' data were acquired. The detail process is described as follows. Twenty healthy subjects (average ages 26.5 ± 3.7 years) wearing the BEAR-H1 were recruited to perform walking on a level surface in the training mode. Each subject performed a series of trials at three kinds of stride frequencies: slow, normal, and fast (0.25, 0.3, 0.4 Hz). Each trial lasted 6 min and 30-s intervals under each of the three walking patterns. Only data were collected for analysis of gait events when subjects adapted their walking styles to the rhythm of the BEAR-H1, and almost 50 strides from each walking pattern were used in the analysis for a total of 150 strides per subject.

For the purpose of the present study, only the outputs of the gyroscope orthogonal to the sagittal plane and the forward accelerometer orthogonal to the coronal plane were retained for analysis. The data of thigh angle θ_z_, thigh angle velocity ϖ_*z*_, forward axial acceleration *a*_*x*_, and the two pressure sensors were logged at 100 Hz on a micro SD card on the PCB. They were sampled at 100 Hz and processed in MATLAB. A digital second-order Butterworth low-pass filtered with a cutoff frequency of 5 Hz was applied off-line to analyze the collected three signals. At present, there were no clear guidelines on the cutoff frequency to be used. The cutoff frequency of 5 Hz was selected for its adequate performance by the experimental verification (Catalfamo et al., 2010).

This protocol was approved by the institutional ethics committee of The Seventh Affiliated Hospital, Sun Yat-sen University, and written informed consent was obtained from all patients.

### Sharing Characteristics and Methods for Gait Event Detection

**Figure 2:**
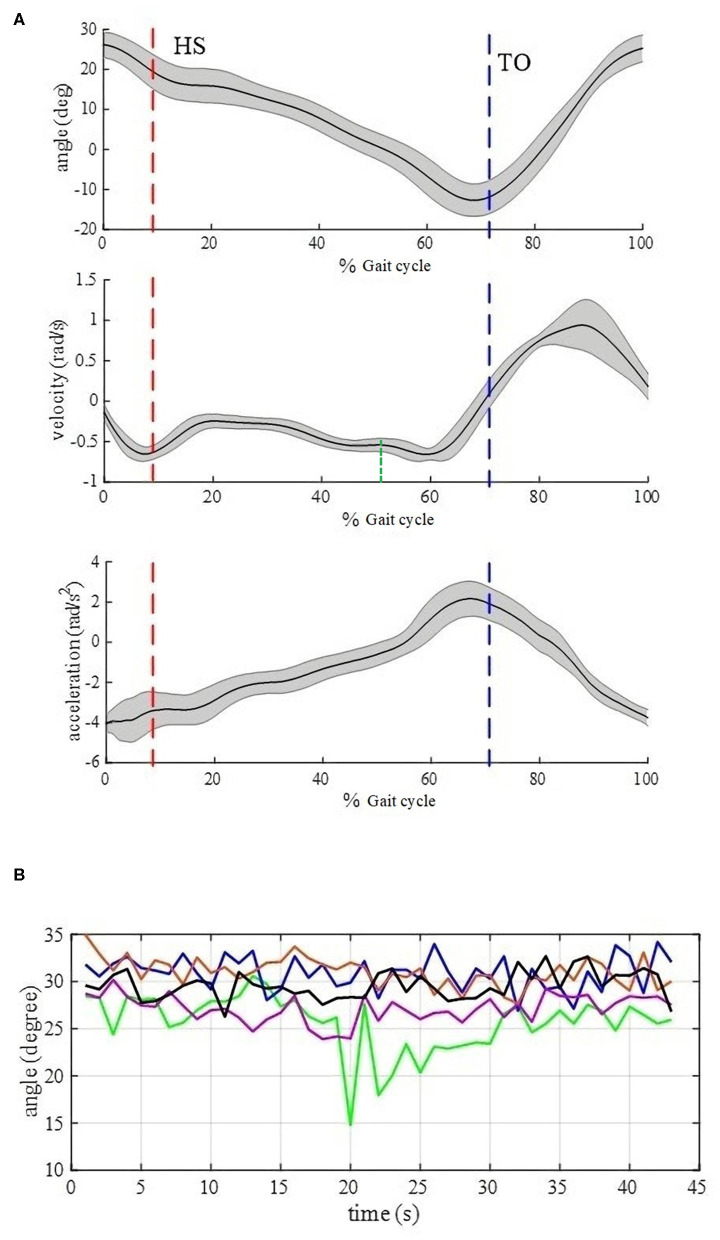
**(A)** Stride parse signals for 43 strides of normal walking by one healthy subject based on foot pressure sensors. The subject wearing the BEAR-H1 walked a level surface at 0.3 Hz stride frequency. The black curve is the mean, and the gray shading shows the standard deviation. **(B)** Thigh angle curves for HS moments. The data points for 43 strides of normal walking at 0.3 Hz are from five healthy subjects wearing the BEAR-H1. The five colors represent five healthy subjects. For instance, the lowest point in the green line means that the thigh angle is 15° when the 20th heel strikes are detected for the “green” subject.

Based on gait data from 20 healthy subjects, at each stride frequency, they share the salient characteristics of time delay relative to thigh angle peaks for HS, low thigh angle for TO, low thigh angular velocity for HS, positive near zero thigh angular velocity for TO, low thigh forward axial acceleration for HS, and high thigh forward axial acceleration for TO. Figure 2A shows these characteristics on one healthy subject based on foot pressure sensors. The subject wearing the BEAR-H1 walked for 43 strides on a level surface at 0.3 Hz stride frequency. The black curve and gray shading show the mean and the standard deviation of 43 gait cycles.

**Figure 3:**
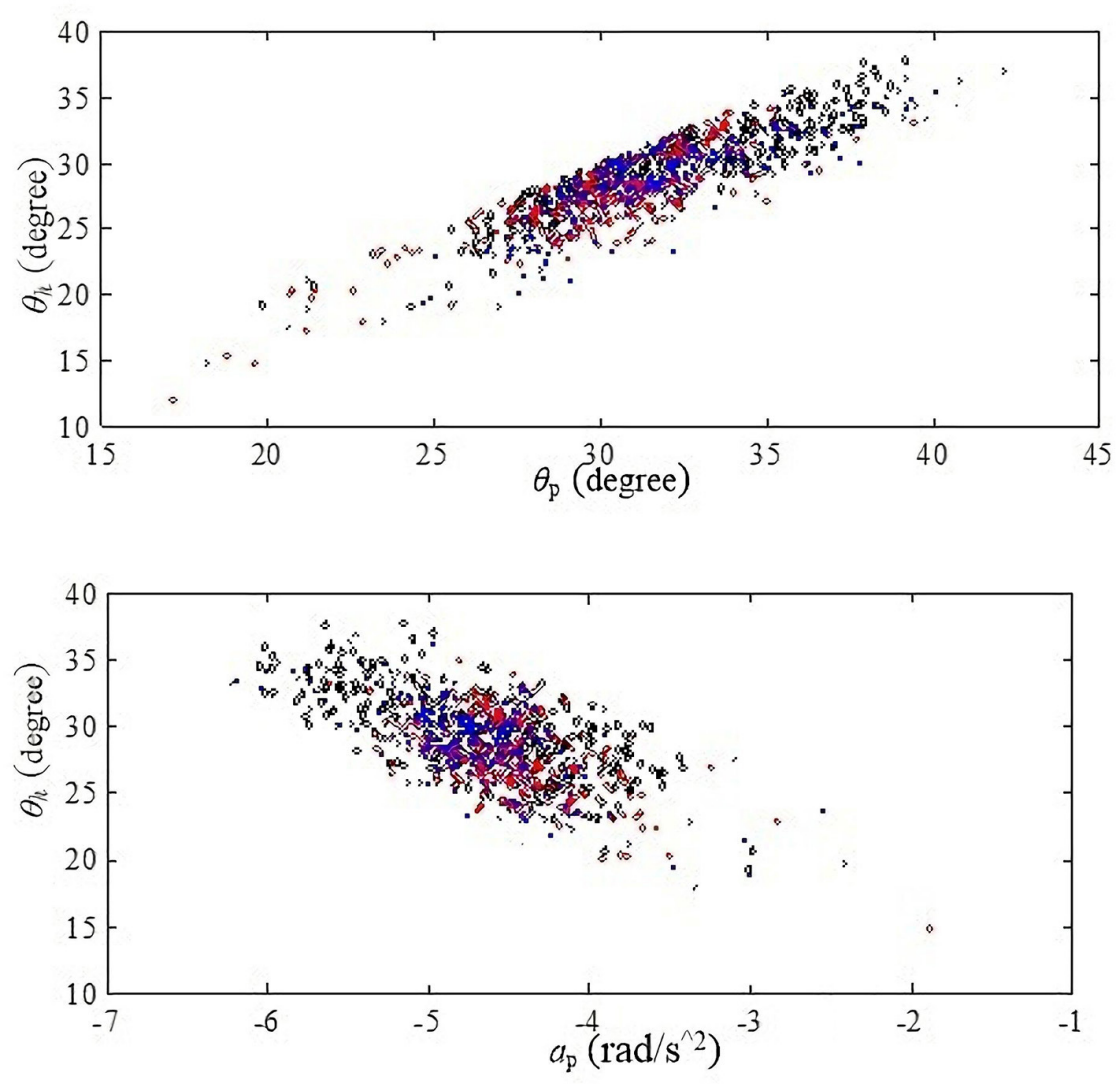
Scatter diagrams. The black color represents the distribution at 0.4 Hz, the red color represents the distribution at 0.3 Hz, and the blue color represents the distribution at 0.25 Hz. Each color has 300 strides of normal walking from six healthy subjects. The above one is the scatter diagram of θ_h_ and θ_p_. The other one is the scatter diagram of θ_h_ and *a*_p_.

For HS, thigh angle difference among five healthy subjects is almost 20° at the same stride frequency, shown in Figure 2B. We find that the fixed threshold (thigh angle) method cannot satisfy the accuracy requirement for HS gait event detection in this case. Besides, the thigh angle decreases monotonously from the peak to the trough. After the thigh angle spike is detected, if thigh angles of HS gait events for different subjects can be obtained accurately in real time, we will make the thigh angle threshold be adjusted adaptively. So we calculated the Pearson correlation coefficient between three features from the thigh angle peak and HS, shown in Table 1. We find that the thigh angle and the forward axial acceleration have strong correlation between the two moments. Therefore, a linear regression model can be used to obtain the thigh angle of HS gait event. The scatter diagrams of the two moments are described in the Figure 3. They show that the three kinds of stride frequencies may share a same linear regression model.

**Table 1:** Pearson correlation coefficient.

	**θ_h_**	**ϖ_h_**	***a*_**h**_**
θ_p_	0.8604	−0.3798	0.5932
ϖ_p_	−0.2017	−0.1262	0.0828
*a_*p*_*	−0.6393	0.0883	−0.7062

*θ_h_, θ_p_ represent thigh angles at HS moment and peak moment, respectively. ϖ_H_, ϖ_p_ represent the angular velocity at HS moment and peak moment, respectively. a_h_, a_p_ represent the forward acceleration at HS moment and peak moment, respectively*.

For HS, the detailed process for gait event detection is described with the following steps:

**Table:** 

**Step 1:** detect thigh angle peak
**if** θ_*t*_ > θ_*t*−1_
**repeat** θ_*t*_, ϖ_*t*_, *a*_*t*_ are sampled
t = *t* + 1
**until** θ_*t*_ < θ_*t*−1_
**end**
get a_*p*_, θ_*p*_, ϖ_*p*_ of thigh angle peak
**Step 2:** obtain the thigh angle threshold for HS
θ_*h*_ = B + *W* × [*a*_*p*_ θ_*p*_ ϖ_*p*_]^*T*^
**Step 3:** detect HS gait event
**if** θ_*t*_ ≤ θ_*h*_
HS gait event is detected
**End**

Where *t* is the current sampling time and *t*-1 is the last sampling time. θ_*t*_, ϖ_*t*_, *a*_*t*_ represent thigh angle, thigh angular velocity, and thigh angular acceleration in current sampling time, respectively. θ_*h*_ represents adaptive thigh angle threshold for HS detection. θ_*p*_, ϖ_*p*_, *a*_*p*_, represents thigh angle, angular velocity thigh, and angular acceleration when the thigh angle reaches the peak. Definition of *B* and *W* can be seen in formula (1) below.

A linear regression model can be described as followes:

(1) $$\begin{array}{l} \text{Y}\left(\text{X}\right)=\text{B}+\text{WX} \end{array}$$

where *X* is the input signal vector, *B* is a one-dimensional constant, and *W* is a 1 × 3 vector of constant coefficients. *Y* is the output: the thigh angle value of the HS moment. For a linear regression model, four parameters are required.

For TO event detection, we apply a fixed thresholding method because there are characteristics for thigh angle θ, angular velocity ϖ, and angular acceleration *a* at TO events. The coordinate axis demonstrated in Figure 1A, where *X, Y, Z* axes are along with the back-to-front, top-to-bottom, and left-to-right direction, respectively. We calculated the mean and standard deviation for thigh angle θ_z_, angular acceleration *a*_*x*_, and angular velocity ϖ_*z*_ at TO events among all strides from 20 healthy subjects wearing the BEAR-H1 and walking at all stride frequencies. Based on the statistical results of −8.31 ± 5.17 for θ_z_, 1.76 ± 1.32 for *a*_*x*_ and 0.32 ± 0.12 for ϖ_*z*_, the value of θ_z_ should be negative while the value of *a*_*x*_ and ϖ_*z*_ should be positive at TO events.

Thus, the algorithm of TO event detection can be described as following:

when

(2) $$\begin{array}{l} \varpi_{z}\left(t\right)\geq \varpi^{*}\text{rad}/\text{s} \end{array}$$

(3) $$\begin{array}{l} \theta_{z}\left(t\right)<\theta^{{\;}^{*}}\text{degree} \end{array}$$

(4) $$\begin{array}{l} a_{x}\left(t\right)>a^{*}\text{rad}/{\text{s}}^{2} \end{array}$$

are satisfied, a TO event is detected.

Where ϖ^*^, θ^*^, a^*^ represent threshold of angular velocity, thigh angle, and angular acceleration, respectively. ϖ_*z*_ *(t)* and θ_*z*_ *(t)* represent angular velocity and thigh angle in current sampling time *t*, along with the *Z-*axis. *a*_*x*_ *(t)* represents angular acceleration in the current sampling time along with the *X-*axis.

The detailed progress of TO detection is described as the following steps:

(1) While HS is detected, the algorithm of TO event detection keeps inactive and waits idly for a period of time *t*_*d*_. (2) Once *t*_*L*_ = *t*_*d*_, the algorithm of TO event detection is activated. (3) Once TO is detected, the algorithm of TO event detection will shut down and wait for the next HS detection; *t*_*L*_ is reset as *t*_*L*_ = 0, where *t*_*d*_ = α*T* and *T* = *t*_*N*_ –* t*_*N*−1_. *T* is time duration of a gait cycle. *t*_*N*_ and *t*_*N*−1_ represent time points of the *N*th and (*N*-1)th occurrences of HS. *t*_*L*_ is the time period that has lasted after HS is detected in the current gait cycle. α is a coefficient that determines how long the algorithm of TO event detection keeps inactive in step 1.

**Figure 4:**
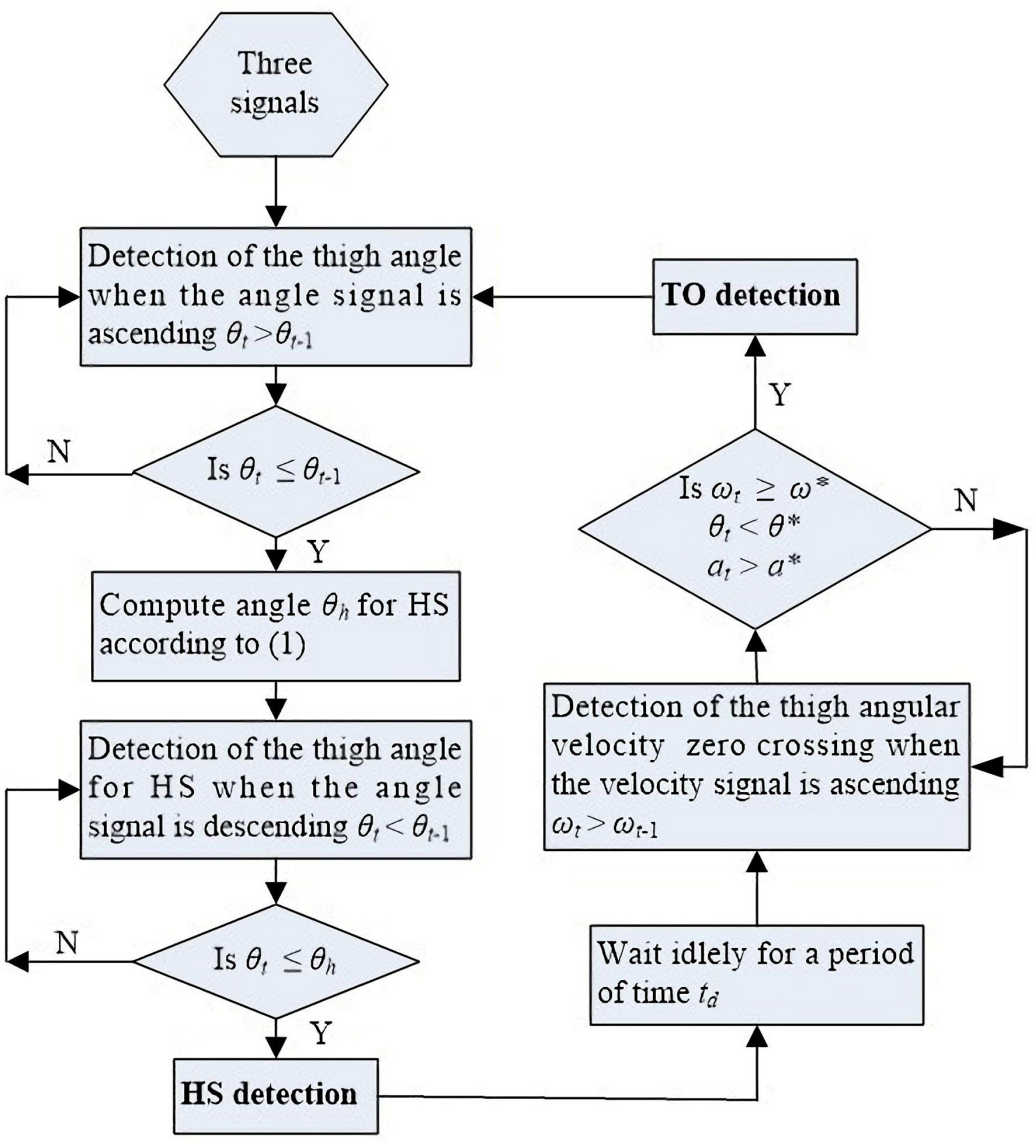
Flowchart of the method used for detection of HS and TO gait events based on the IMU's three signals.

For TO, three threshold parameters are required. The angular velocity is chosen to be the limiting threshold for its signal feature directly leads to TO gait event occurrence. The flowchart of the proposed method used for detection of HS and TO gait events is described in detail in Figure 4.

### Model Training and Parameter Selection

For HS detection, the data from 20 healthy subjects were classified into training and testing sets for linear regression models, and five different train:test subject ratios were adopted, ranging from 9:1 to 5:5. There is a linear regression model in each train:test ratio. Five linear regression models are obtained based on the 0.25 Hz data set, 0.30 Hz data set, 0.40 Hz data set, and all data set, respectively.

For TO detection, threshold parameters were selected for gait event detection based on observing gait cycle parsed signals of thigh angle, angular velocity, and forward axial acceleration. The proposed method thresholds and parameter determined are θ^*^ = 0, ϖ^*^ = 0.2, a^*^ = 0, and α = 0.4.

Based on the statistics of 20 healthy subjects, we obtain results of −8.31 ± 5.17 for thigh angle θ_*z*_, 0.32 ± 0.12 for angular velocity ϖ_*z*_, and 1.76 ± 1.32 for angular acceleration *a*_*x*_ at TO events. ϖ^*^ = 0.2 locates at the lower boundary of standard deviation (mean minus standard deviation) of angular velocity in order to suit formula (2). a^*^ = 0 locates at below the lower boundary (mean minus standard deviation) of the standard deviation of angular acceleration in order to suit different subjects in formula (4). θ^*^ = 0 locates at above the upper boundary of the standard deviation (mean plus standard deviation) of the thigh angle in order to suit different subjects in formula (3).

HS events are generally defined at 0% of *T*, and TO events are at about 58% of *T* (Perry, 2010), where *T* is defined in Section Sharing Characteristics and Methods for Gait Event Detection. Thus, α can be set as 0.58 maximally, which means that the algorithm of TO detection waits for 58% of *T* after HS events occur. Any higher values exceeding 0.58 may lead to the failure of TO detection because the timing of TO has been lapsed when the algorithm is activated. However, we set α = 0.4, giving enough time for the TO detection algorithm in advance in order to ensure that our algorithm works for different subjects because some subjects walk with earlier TO events. Meanwhile, α = 0.4 can avoid potentials of misjudgment. Referring to Figure 2A, there is fluctuation between HS and TO in the angular velocity curve and any up-trends may lead to a sharp rise of angular velocity, causing satisfaction of formula (2) and a wrong TO detection. α = 0.4 locates at the green dotted line, and there is only an up-trend of angular velocity after this line, which reduces the potential of TO misjudgment.

Two performance metrics were number of detected gait event (frequency) and percentage stride error of gait event detection (temporal) as compared to foot pressure sensors. For HS, the test set was used to assess the two performance metrics. For TO, whole subjects walking at three kinds of stride frequencies were used to assess the performance metrics. First, the strides were parsed correctly by data from foot pressure sensors as a reference and then calculated the averages and standard deviations of errors of the two performance metrics from the proposed method and the conventional method for gait event detection.

## Experiments

The number of detected gait events (frequency) and the percentage of gait cycle error of gait event (temporal) were calculated based on the proposed method and the conventional thresholding method. Detailed descriptions are described subsequently. For frequency error, a positive number represents extra gait event detection, and a negative number means miss of a gait event. For temporal error, a positive number represents early detection, and a negative number means delayed detection.

### Healthy Subjects

**Figure 5:**
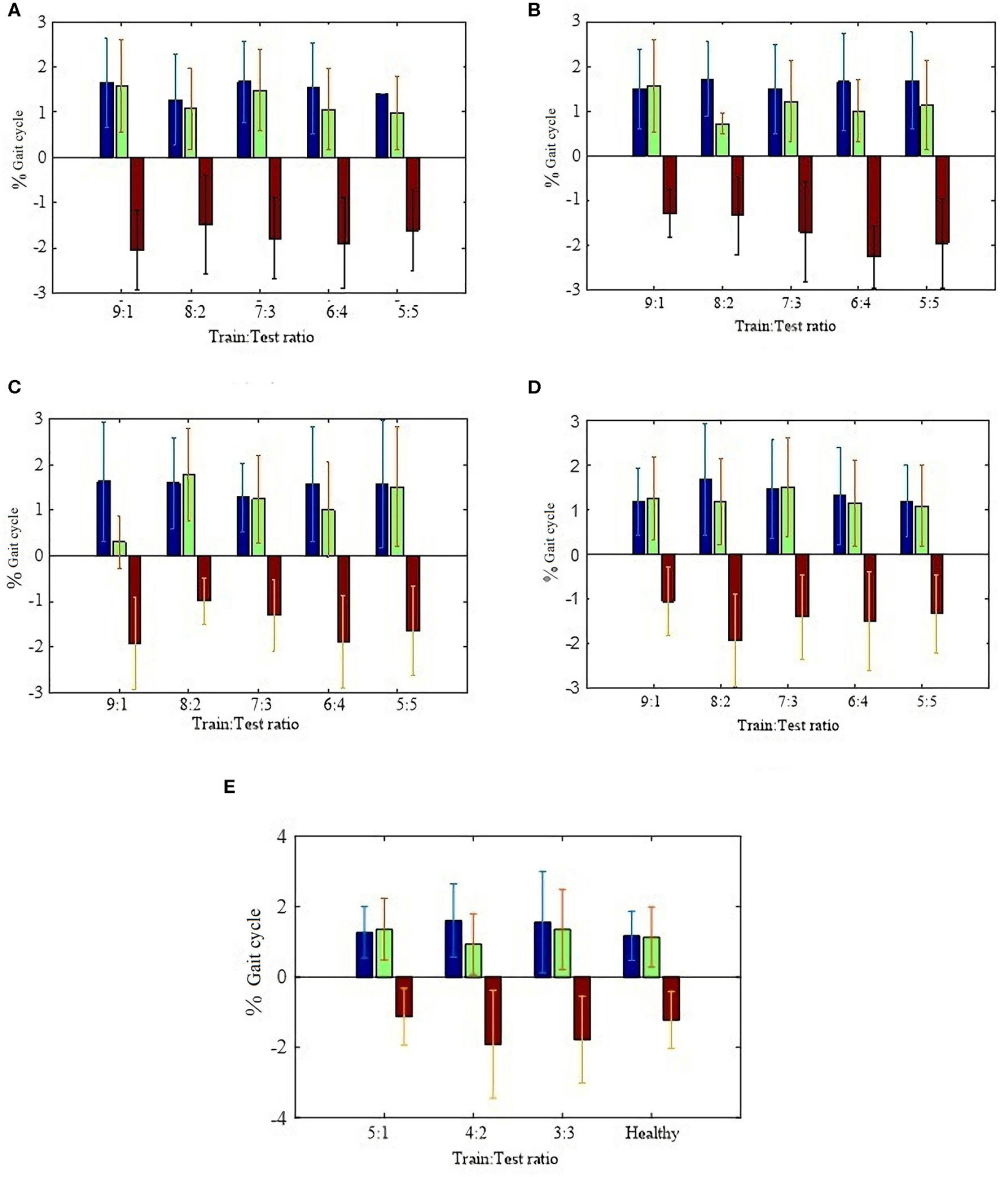
Blue, green, and red represent mean absolute deviation, mean positive deviation, and mean negative deviation. **(A)** HS % gait cycle error at 0.25 Hz stride frequencies; **(B)** HS % gait cycle error at 0.30 Hz stride frequencies; **(C)** HS % gait cycle error at 0.40 Hz stride frequencies; **(D)** HS % gait cycle error at all kinds of stride frequencies; **(E)** Patients HS % gait cycle error at all kinds of stride frequencies.

For HS, performance comparisons of each train:test ratio are summarized in Figure 5. We find that the frequency performance for HS gait event detection does not improve significantly when the training set becomes bigger. Besides, the accuracy also does not improve significantly when the model from a data set of same stride frequency or from a data set of all stride frequencies is applied to estimate HS gait event detections, respectively, shown in Figure 5, Table 2 as mean ±1 standard deviation (7:3 ratio). Thus, the proposed method for HS gait event detection adopts the linear regression (LR) model from the data set of all kinds of stride frequencies based on a 7:3 train:test ratio. Error comparisons between the proposed method and the conventional method are shown in Table 3 as mean ±1 standard deviation. The evaluation index is based on the mean absolute deviation (MAE). For TO, based on the set of observable fixed thresholds, temporal, and frequency performances for all subjects at three kinds of stride frequencies are also described in Table 3. Temporal absolute errors at different stride frequencies based on the proposed method are 1.3 ± 1.1% gait cycle for HS and 1.8 ± 1.1% gait cycle for TO. The proposed method can detect all HS/TO gait events precisely. However, the conventional thresholding method misses HS gait event detections when a set of fixed thresholds is used.

**Table 2:** HS results from different stride frequency training data.

	**0.25 Hz**	**0.3 Hz**	**0.4 Hz**	**All stride frequency**
	**data**	**data**	**data**	**data**
Temporal	1.6 ± 1.1	1.5 ± 1.0	1.6 ± 1.2	1.3 ± 1.1
Frequency	0	0	0	0

**Table 3:** Healthy results from healthy training.

	**Performance**	**Proposed method**	**Conventional method**
Temporal	HS error (%gait cycle)	1.3 ± 1.1	1.7 ± 0.6
	TO error (%gait cycle)	1.8 ± 1.1	1.8 ± 0.6
Frequency	HS error (%gait cycle)	0	−5.6
	TO error (%gait cycle)	0	0

### Patients

In order to verify the feasibility of the proposed method for patients with hemiplegia, six patients with hemiplegia (average ages 45.5 ± 8.7 years) wearing the BEAR-H1 were recruited to walk on a level surface. For patients' safety, they walked at their preferred stride frequencies in the intelligent mode of BEAR-H1. All patients signed an informed consent document prior to participating. The processes of data acquisition and processing are same as healthy subjects.

For TO gait event detection, the threshold parameters are θ^*^ = 0, ϖ^*^ = 0.15, *a* ^*^ = 0, and α = 0.4. Similar to the selection of θ^*^, ϖ^*^, *a* ^*^, and α for healthy subjects in Section Model Training and Parameter Selection, these parameters are identified based on statistics from all hemiplegic subjects. Statistical results are −7.68 ± 4.36 for θ_*z*_, 0.26 ± 0.11 for ϖ_*z*_, and 1.27 ± 1.08 for *a*_*x*_ at TO events. ϖ^*^ = 0.15 locates at the lower boundary of standard deviation (mean minus standard deviation) of angular velocity in order to suit formula (2). a^*^ = 0 locates at below the lower boundary (mean minus standard deviation) of the standard deviation of angular acceleration in order to suit different subjects in formula (4). θ^*^ = 0 locates at above the upper boundary of the standard deviation (mean plus standard deviation) of the thigh angle in order to suit different subjects in formula (3). α = 0.4 avoids the fluctuation between HS and TO in angular velocity, which may lead to the TO misjudgment.

For HS gait event detection, three different train:test subject ratios are used, ranging from 5:1 to 3:3. Performance comparisons of each train:test ratio are summarized in Figure 5E. Performance based on the LR model from the healthy subject data set is also described in Figure 5E. We also find that the performance gap between them is not big. Thus, the proposed method for HS gait event detection of patients with hemiplegia still adopts the LR model and error comparisons between the proposed method and the conventional method are shown in Table 4 as mean ±1 standard deviation.

**Table 4:** Patients results from healthy training.

	**Performance**	**Proposed method**	**Conventional method**
Temporal	HS error (%gait cycle)	1.2 ± 0.7	1.9 ± 0.3
	TO error (%gait cycle)	1.3 ± 0.7	1.8 ± 1.1
Frequency	HS error (%gait cycle)	0	−4.2
	TO error (%gait cycle)	0	0

## Discussion

Our study provides an adaptive thresholding method for HS detection based the LR model at various stride frequencies. It can realize threshold accurate adjustment. For TO, different subjects walking at various stride frequencies share a same characteristic; that is, angular velocity increases above ϖ^*^ rad/s^2^, thigh angle is <θ^*^ degree (thigh neutral positions), and forward axial acceleration is higher than a^*^ rad/s^2^. When a sample meets these three conditions, a TO gait event is detected.

**Figure 6:**
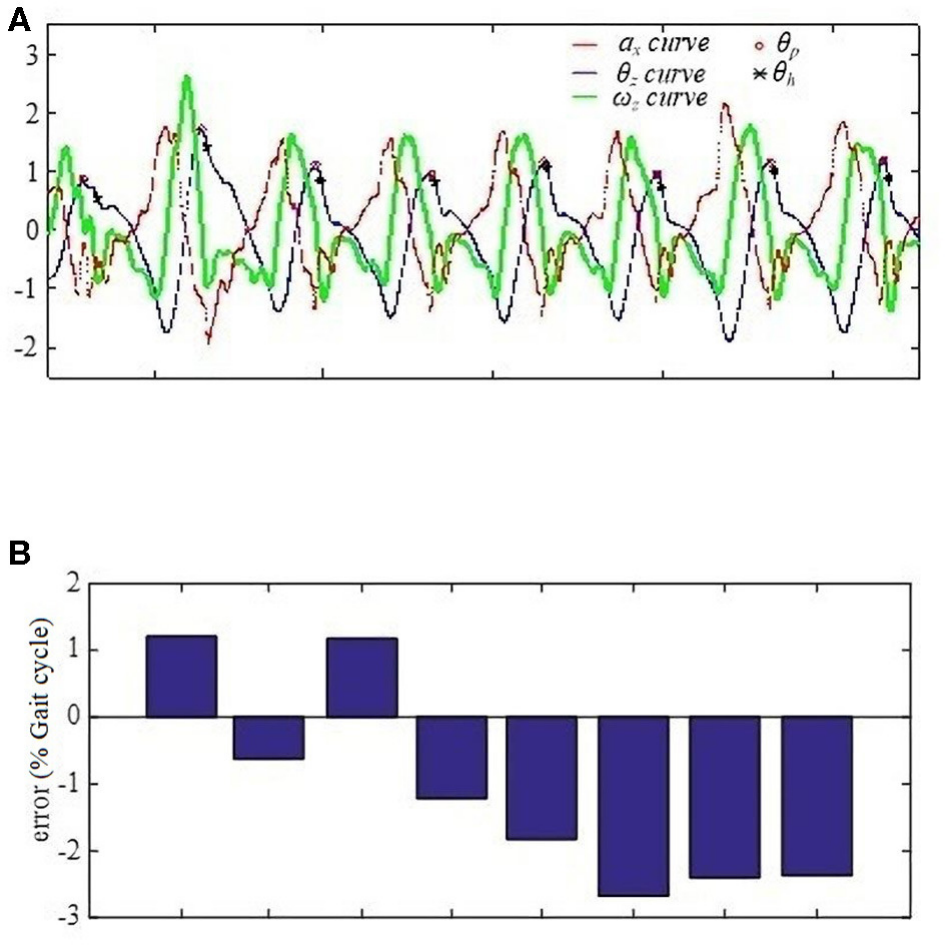
HS % gait cycle error from one subject. **(A)** The three signal normalized values when the subject walks continuously on a level surface at 0.3 Hz stride frequency. x-axis represents the time, and y-axis is the normalized values. **(B)** Timing percentage errors of the eight gait cycles. y-axis is cycle number.

For HS, because stride lengths affect thigh angle spikes, when a subject wearing the BEAR-H1 walks at one kind of stride frequency, thigh angles may change within a relatively large range. In this case, the model can still detect HS gait events within 3% gait cycle errors and with −1.2 ± 1.6% as shown in Figure 6 although the most recent research reports gait cycle error of −2.1 ± 1.0% at HS detection on the knee exoskeleton (Xu et al., 2019). Another gait event detection (Schicketmueller et al., 2019) conducted on low limb exoskeleton robots (Locomat and Lyra) obtain the highest accuracy, 98.1 ± 5.2%, but they did not evaluate the time delay problem for their results. Besides, the LR model can accurately detect HS gait events for healthy subjects walking at three kinds of stride frequencies and hemiplegic patients walking at their preferred stride frequency. The frequency errors are 1.3 ± 1.1% gait cycle for healthy subjects (shown in Table 3) and 1.2 ± 0.7% gait cycle for patients (shown in Table 4), respectively. They are less than frequency errors of the conventional method (Ledoux, 2018). Thus, we believe that the proposed method based on the LR model for HS detection is quite robust.

For HS gait event detection, the frequency error for patients (1.2 ± 0.7% gait cycle) is less than the frequency error for healthy subjects (1.3 ± 1.1% gait cycle). The reason is that patients with hemiplegia have slightly different gait characteristics due to a reduced range of motion relative to healthy subjects as well as reduced lower limb functionality and performance.

Foot pressure sensors used here are considered as a convenient way to generate the labeled data sheet for HS and TO gait event detection. The accuracy of gait detection based on foot pressure sensors may be questioned. There will be irreversible physical damage for foot switches after the maximum number of uses, and this will be inconvenient for long-term use. For users who have suffered from stroke, most of them have strephenopodia, which may cause uneven pressure on the sole and lead to the failure of gait event detection. In this paper, judgments for HS and TO gait events are based on downward and upward trends of foot pressure sensors but not on their actual values.

Our method shows robustness on gait event detection with negligible time delay. In our future work, we plan to implant our method into different devices (BEAR-H1 exoskeleton) because our previous experiments are only conducted on one device. Meanwhile, more hemiplegic subjects will be included in our research to optimize our method for hemiplegic users.

## Conclusion

It is possible to accurately detect HS and TO gait events for both healthy subjects and patients with hemiplegia using one IMU sensor mounted on the thigh of gait rehabilitation robots. The method can detect HS and TO gait events accurately, and timing errors are within an average 2% gait cycle. For HS gait event detection, first, the proposed method based on the LR model can be applied for healthy subjects and patients with hemiplegia. Second, the LR model can be used at various stride frequencies. Finally, the LR model can more accurately detect HS gait event for patients with hemiplegia than healthy subjects. In the future, we will attempt to detect more gait events based on the proposed method.

## Data Availability Statement

The datasets generated for this study are available on request to the corresponding author.

## Ethics Statement

Ethical review and approval was not required for the study on human participants in accordance with the local legislation and institutional requirements. The patients/participants provided their written informed consent to participate in this study.

## Author Contributions

JY proposed the prototype of the gait event detection method. HW and JY modified and finalized the design of this method. HW designed the experiment and drafted and revised the manuscript. LW conducted the most of experiments and data analysis on hemiplegic patients. JL was in charge of the experiments for the rest hemiplegic patients and relevant data analysis. YZ and GC conducted experiments for parts of healthy subjects and analysis the data of healthy subjects. CW and XL conducted experiments for the rest of healthy subjects. YX and QH provided theoretical guidance for the proposed method and experiments design and made the final approval of the submitted version.

## Conflict of Interest

JY, HW, and GC were employed by the company Shenzhen MileBot Robotics Co., Ltd. The remaining authors declare that the research was conducted in the absence of any commercial or financial relationships that could be construed as a potential conflict of interest.
